# Viral and bacterial etiologies of community-acquired acute lower respiratory infections among hospitalized Cambodian patients

**Published:** 2011-01-10

**Authors:** Sirenda Vong, Bertrand Guillard, Laurence Borand, Sophie Goyet, Blandine Rammaert, Sopheak Hem, Sek Mardy, Philippe Cavailler, Vantha Te, Patrich Lorn Try, Philippe Buchy

**Affiliations:** 1 Epidemiology and Public Health Unit, Institut Pasteur in Cambodia, Phnom Penh, Cambodia; 2 Biomedical Laboratory, Institut Pasteur in Cambodia, Phnom Penh, Cambodia; 3 Virology Unit, Institut Pasteur in Cambodia, Phnom Penh, Cambodia; 4 Pediatric Department, Donkeo Provincial Hospital, Takeo, Cambodia; 5 Pediatric Department, Provincial Hospital, Kampong Cham, Cambodia

## 

Community-acquired acute lower respiratory infections (ALRI) remain a major public health problem, particularly in tropical and low/middle-income countries. There is still a paucity of data regarding viral and bacterial etiologies. Beginning in April 2007, we enrolled all-aged patients hospitalized with acute respiratory symptoms excluding known tuberculosis and positive HIV serology in Cambodia. Etiology was assigned based on laboratory data from direct sputum examination, blood and sputum cultures and nasopharyngeal swabs. Diagnosis of ALRI was determined from medical charts’ reviews and interpreting chest radiographs by expert pulmonologists. Severe ALRI case (SARI) was defined on the basis of oxygen saturation, respiratory rates according to age and severe clinical symptoms.

During April 2007 - December 2009, 2,824 patients presented with ALRI on admission including 948 (34%) in the <5 year-age groups. Of the 948 ALRI patients aged <5 years, 43.7% were diagnosed with parenchymal involvement (PI), 0.9% with pleurisy alone and 57.2% bronchi involvement (BI). In the >5 year-olds, the proportions were different: 56.4% PI, 38.1% BI and 12.9% pleurisy.

A total of 821 (28.9%) SARI were identified of which 639 (77.8%) aged <5 years. SARI accounted for 62% of PI in the <5 year-olds and 12% in the >5 year-olds. SARI was also frequently found in BI (71%) among <5 year-old children and significantly less frequent in older children and adults (8%).

Specimens for bacteriology testing was only available in 1,004 patients including 14 who aged <5 years, 30 in the 5 – 17 year age group and 960 (95.6%) among adults. In the 14 <5 year-old children, 12 bacteria were identified including *Burkholderia pseudomallei*, 2 *Streptococcus pneumoniae*, 2 *Haemophilus influenzae,* 2 *Klebsiella pneumoniae* and 4 acid fast-bacilli (AFB); in the 5-17 year age group, there were 4 AFB, 4 *H. influenzae* and 4 *S. pneumoniae*. Of the 960 adults, etiology was found in 525 (64.7%) ones of which the most commonly identified bacteria were AFB (48.8%), *H. influenzae* (21.1%) and *S. pneumoniae* (9.1%) followed by *K. pneumoniae* (7.8%) and *B. pseudomallei* (5.6%). Of the 70 SARI cases for which specimens were available, the most frequently found bacteria were AFB (18.6%), *B. pseudomallei* (10.0%), *H. influenzae* (8.6%), and *K. pneumoniae* (7.1%). Viral identification was possible in 927 (30.1%) of the 3,083 patients’ nasopharyngeal swabs (57.7% in <5 year-olds, 15.5% in adults). The three most frequently identified viruses were rhinoviruses (42.3%), RSV (29.1%), and seasonal Influenza A & B (7%).

This is the first report of the etiology of community-acquired ALRI in Cambodia – a tropical/low-income country. Our results indicated that the etiology profile in Cambodia was similar to that of other Southeast Asian countries.

Supported by the French Development Agency through the Surveillance and Investigation of Epidemic Situations in Southeast Asia (SISEA) project.

